# Validity of the Chronic Airways Assessment Test (CAAT) in asthma, asthma+COPD and COPD in NOVELTY

**DOI:** 10.1183/23120541.01359-2024

**Published:** 2025-07-21

**Authors:** Paul W. Jones, Erin L. Tomaszewski, Laura Belton, Pierre-Régis Burgel, Rod Hughes, Christina Keen, Barry J. Make, Alberto Papi, Hana Müllerová, Helen K. Reddel, for the NOVELTY Scientific Community and the NOVELTY study investigators

**Affiliations:** 1 Global Respiratory Franchise, GSK, Brentford, UK; 2 Patient Centered Science BioPharmaceuticals Medical, AstraZeneca, Gaithersburg, MD, USA; 3 Biostatistics, BioPharmaceuticals Medical, AstraZeneca, Cambridge, UK; 4 Department of Respiratory Medicine, Cochin Hospital, Assistance Publique-Hôpitaux de Paris, Paris, France; 5 Université Paris Cité, Institut Cochin, Inserm U1016, Paris, France; 6 Research and Early Development, Respiratory and Immunology, Clinical, BioPharmaceuticals R&D, AstraZeneca, Cambridge, UK; 7 Research and Early Development, Respiratory and Immunology, Clinical, BioPharmaceuticals R&D, AstraZeneca, Gothenburg, Sweden; 8 National Jewish Health and University of Colorado Denver, Denver, CO, USA; 9 Respiratory Medicine Unit, Department of Translational Medicine, Università di Ferrara, Ferrara, Italy; 10 Respiratory Evidence Strategy, BioPharmaceuticals Medical, AstraZeneca, Cambridge, UK; 11 The Woolcock Institute of Medical Research and Macquarie Medical School, Faculty of Medicine, Health and Human Sciences, Macquarie University; The University of Sydney; and Sydney Local Health District, Sydney, Australia; 12 P.W. Jones and E.L. Tomaszewski contributed equally

## Abstract

**Background:**

To assess health status in respiratory diseases, the Chronic Airways Assessment Test (CAAT) was adapted from the COPD Assessment Test (CAT) by replacing COPD-specific wording. It has demonstrated good psychometric properties in asthma and/or COPD. This cross-sectional analysis evaluated how CAAT scores are associated with clinical characteristics in patients with asthma and/or COPD.

**Methods:**

Using baseline NOVELTY data (NCT02760329) for patients with physician-assigned asthma and/or COPD, linear regression models were implemented to assess the association between CAAT score (range 0–40; higher scores indicating worse health status) and physician-assessed severity, lung function, modified Medical Research Council dyspnoea grade, Respiratory Symptoms Questionnaire score and, for asthma and asthma+COPD, symptom control assessed by Asthma Control Test score.

**Results:**

Among 7828 patients (asthma: 4138; asthma+COPD: 991; COPD: 2699), CAAT score was lower in patients with asthma (mean±sd 14.0±8.5) *versus* patients with asthma+COPD (17.2±8.6) or COPD (17.0±8.3), indicating better health status in asthma. Associations between CAAT score and clinical characteristics were similar across diagnostic groups (interaction p-values >0.01), with higher CAAT scores associated with more respiratory symptoms, greater exercise limitation due to breathlessness, lower lung function, worse physician-assessed severity and (in asthma+COPD) with worse asthma symptom control. CAAT scores among those with asthma were lower *versus* other diagnostic groups by physician-assessed severities. Findings were similar when adjusting for age and for age, sex and smoking status.

**Conclusion:**

The CAAT demonstrated consistent cross-sectional validity across asthma and/or COPD, making it applicable for assessing health status in these conditions in clinical practice and research.

## Shareable abstract

**The CAAT demonstrates consistent cross-sectional validity across asthma and/or COPD, making it suitable for assessment of health status in adults in research and clinical practice** https://bit.ly/44S5sww

## Introduction

Living with asthma and/or COPD can have a major impact on a person's life [1, 2]. Using patient-reported outcomes (PROs) to directly capture the patient's perspective regarding their health status and well-being is important to evaluate disease impact and direct therapeutic choices in routine clinical practice [3]. PROs can also capture essential information about disease stability/progression, future exacerbation risk and response to changes in disease management [4, 5].

Most PROs used in clinical practice for patients with respiratory disease pose challenges in that they have been developed for specific diagnoses, do not focus primarily on health status and/or are often complex and time-consuming to complete and score. The COPD Assessment Test (CAT) is a short, simple PRO that is used to measure health status in COPD, both in clinical practice and in research settings [6–9], and is recommended by the Global Initiative for Chronic Obstructive Lung Disease [3]. However, there is no equivalent, short, simple instrument available to measure health status in asthma or other airways diseases. Measures such as the Asthma Control Test (ACT) and the Asthma Control Questionnaire (ACQ), focus on symptom control rather than the impact on patient health [10–12] and do not include questions about symptoms such as sputum production, which is common in asthma [4]. Further, none of these asthma-specific questionnaires have been validated in patients with both asthma and COPD. The St George's Respiratory Questionnaire (SGRQ) has been developed and validated to measure health status in both patients with asthma and COPD [13] and has been validated as an outcome measure in patients with severe asthma [14, 15]. However, it is time-consuming to complete and score, comprising 50 items with 76 weighted responses [13, 16]. The 20-item Airways Questionnaire was developed and validated as a shorter and simpler alternative to the SGRQ in both asthma and COPD, but completion may still be somewhat time-consuming [17, 18].

To overcome the challenges associated with existing questionnaires, we derived the Chronic Airways Assessment Test (CAAT) from the CAT to create a short, simple, standardised, eight-item health status measure for use across respiratory diseases [19]. The CAAT was modified from the CAT [6] with the permission of the copyright holder; the only changes were replacement of COPD-specific wording in the title and introduction with “chronic airways” and “pulmonary disease”, respectively. The CAAT comprises the same eight items, responses and scoring system as the CAT (supplementary figure 1).

The CAAT has demonstrated good cross-sectional psychometric properties in a random sample of patients with asthma, asthma+COPD and COPD from NOVELTY (NOVEL observational longiTudinal studY; NCT02760329) [19]. This quantitative analysis showed that patients with asthma and COPD responded to the individual CAAT items in a similar way [19]. It has been shown to correlate with the SGRQ (high correlation reflected by R^2^ >0.86 across all diagnostic groups) and the EuroQoL five-dimensions five-level visual analogue scale (EQ-5D-5L VAS) [19], demonstrating its potential suitability for use across respiratory diseases. Due to its brevity and its inclusion of a range of different factors that may impact health status in people with airways disease, the CAAT could be beneficial for use in clinical practice and for clinical studies to assess patient-perceived health status and the effect of treatment interventions on this measure [19]. Routine use across clinical settings of a single questionnaire that is applicable to both asthma and COPD will be more convenient and practical for clinicians, and may facilitate wider uptake of assessment of health status, including in patients with features of both asthma and COPD, or in whom the diagnosis of asthma and/or COPD is suspected [20, 21].

The present analysis was designed to test the validity of the CAAT by examining its cross-sectional association with clinical characteristics in patients enrolled in NOVELTY with physician-assigned diagnoses of asthma, asthma+COPD or COPD, and to assess whether these associations differed by diagnostic group.

## Material and methods

### NOVELTY study population

NOVELTY is a global, prospective, observational study of 11 192 patients with a physician-assigned diagnosis of asthma and/or COPD, conducted across 18 countries. The NOVELTY study design and patient population have been reported previously [20, 22, 23]. Briefly, NOVELTY enrolment was stratified by physician-assigned diagnosis or suspected diagnosis (asthma, asthma+COPD or COPD) and physician-assessed severity (mild, moderate or severe), to avoid the selection bias observed in regulatory studies and to allow sufficient numbers for sub-group analysis [22, 24]. To reflect real-world populations, no diagnostic or severity criteria were specified to physicians [20, 22]. For patients with asthma+COPD, physician-assessed severity was the higher of the separate severity classifications for asthma and COPD.

### CAAT properties

Each item is scored 0–5 with a total score range of 0–40. Higher CAAT scores indicate worse health status. The CAAT is copyrighted by GSK, but free for use by clinicians and academics with permission. CAAT activities are monitored by a Supervisory Council, which includes independent experts, on the Global Allergy and Airways Patient Platform [25].

### Clinical characteristics

Baseline clinical characteristics were selected for analysis based on their relevance as assessments of disease severity and impact in asthma, COPD or both conditions. Physician-reported measures recorded by the physician at the baseline visit included physician-assessed severity (mild, moderate, severe) and post-bronchodilator forced expiratory volume in 1 s (FEV_1_ % pred) (based on Global Lung Function Initiative multi-ethnic reference equations) [26]. The modified Medical Research Council (mMRC) dyspnoea scale [27] was also recorded during the patient visit. The mMRC dyspnoea scale is graded from 0 to 4, with higher grades indicating greater activity limitation due to dyspnoea. Other patient-reported measures were separately completed by the patient online or by telephone within 21 days after the baseline visit. In addition to the CAAT, these included the Respiratory Symptoms Questionnaire (RSQ) [28], the SGRQ [13] and, for patients with asthma or asthma+COPD, the ACT [11]. The RSQ is scored from 0 to 16, with higher scores indicating worse respiratory symptoms, the SGRQ is a 50-item questionnaire scored on a 0–100 scale, with higher scores indicating worse health status, and the ACT is scored from 5 to 25, with higher scores indicating better symptom control.

### Statistical analysis

All available patients from NOVELTY who completed the baseline CAAT questionnaire and who had no missing baseline data for physician-assigned diagnosis and physician-assessed severity were included in the analysis.

Linear regression models were implemented using baseline data to assess the association between CAAT score (as a continuous outcome measure) and each clinical characteristic separately as a covariate along with diagnostic group. Post-bronchodilator FEV_1_% pred, RSQ score and ACT score were analysed as continuous variables, whilst physician-assessed severity and mMRC dyspnoea grade were analysed as categorical variables. To test whether the association between CAAT score and each clinical characteristic differed between diagnostic groups, an interaction term was included in each model and interaction p-values were generated. Marginal trends of the continuous variables and contrasts of each level for the categorical variables were estimated and reported along with the corresponding standard errors and 95% confidence intervals. Statistical differences of the pairwise comparisons were evaluated using two-sided p-values.

Two sensitivity analyses were also implemented for each clinical characteristic model, namely one adjusting for age as an additional covariate and another adjusting for age, sex and smoking status as additional covariates.

## Results

### Patient baseline characteristics

In total, 7828 patients from 18 countries were included in this analysis (mean±sd age 59.8±sd 14.7; female 52.4%), of whom 4138 had physician-assigned asthma, 991 had physician-assigned asthma+COPD and 2699 had physician-assigned COPD (supplementary figure 2). Baseline demographics and clinical characteristics are reported in table 1 and supplementary table 1. Patients with a diagnosis of asthma were younger, more likely to be female and a never-smoker and had higher lung function than those with asthma+COPD or COPD.

**TABLE 1:** Patient baseline demographics and clinical characteristics by physician-assigned diagnostic group

	Asthma (N=4138)	Asthma+COPD (N=991)	COPD (N=2699)	Total (N=7828)
**Age in years, mean±sd**	53.7±15.8	65.2±9.9	67.2±9.1	59.8±14.7
**Female, n (%)**	2625 (63.4)	449 (45.3)	1028 (38.1)	4102 (52.4)
**Region, n (%)**				
Europe	1739 (42.0)	477 (48.1)	1221 (45.2)	3437 (43.9)
Australia and Canada	724 (17.5)	191 (19.3)	426 (15.8)	1341 (17.1)
Japan and Korea	770 (18.6)	161 (16.2)	229 (8.5)	1160 (14.8)
USA	541 (13.1)	124 (12.5)	404 (15.0)	1069 (13.7)
Latin America	364 (8.8)	38 (3.8)	419 (15.5)	821 (10.5)
**Body mass index in kg·m^−2^**				
Patients with data, n	3837	947	2536	7320
Mean±sd	28.1±6.7	28.6±6.6	27.6±6.3	28.0±6.6
**Years since asthma diagnosis**				
Patients with data, n	3981	946	NA	4927
Mean±sd	19.2±17.6	21.3±21.5	NA	19.6±18.4
**Years since COPD diagnosis**				
Patients with data, n	NA	951	2637	3588
Mean±sd	NA	7.2±7.6	7.9±8.4	7.7±8.2
**Physician-assessed severity^#^, n (%)**				
Mild	1485 (35.9)	165 (16.6)	750 (27.8)	2400 (30.7)
Moderate	1456 (35.2)	440 (44.4)	825 (30.6)	2721 (34.8)
Severe	1197 (28.9)	386 (39.0)	1124 (41.6)	2707 (34.6)
**Smoking status**				
Patients with data, n	4133	988	2695	7816
Current smoker, n (%)	306 (7.4)	232 (23.5)	723 (26.8)	1261 (16.1)
Former smoker, n (%)	1299 (31.4)	648 (65.6)	1795 (66.6)	3742 (47.9)
Never smoked, n (%)	2528 (61.2)	108 (10.9)	177 (6.6)	2813 (36.0)
**Pack-years of smoking**				
Patients with data, n	4138	991	2699	7828
Mean±sd	6.4±15.0	31.6±29.0	43.7±39.0	22.4±32.4
**Post-bronchodilator FEV_1_ (% pred)**				
Patients with data, n	3384	831	2261	6476
Mean±sd	86.9±20. 6	68.4±21.8	61.0±23.2	75.5±24.8
**Post-bronchodilator FEV_1_/FVC ratio**				
Patients with data, n	3444	853	2308	6605
Mean±sd	0.740±0.118	0.588±0.147	0.558±0.161	0.657±0.164
**CAAT score^¶^**				
Patients with data, n	4138	991	2699	7828
Mean±sd	14.0±8.5	17.2±8.6	17.0±8.3	15.4±8.6
**mMRC dyspnoea grade ≥2^+^**				
Patients with data, n	3982	957	2636	7575
Yes, n (%)	803 (20.2)	407 (42.5)	1367 (51.9)	2577 (34.0)
**SGRQ score^§^**				
Patients with data, n	4062	975	2659	7696
Mean±sd	29.9±20.9	40.0±22.1	41.6±21.7	35.2±22.1
**Overall health status**				
Patients with data, n	4057	974	2672	7703
Very good, n (%)	448 (11.0)	43.0 (4.4)	143 (5.4)	634 (8.2)
Good, n (%)	1680 (41.4)	303 (31.1)	771 (28.9)	2754 (35.8)
Fair, n (%)	1556 (38.4)	453 (46.5)	1287 (48.2)	3296 (42.8)
Poor, n (%)	325 (8.0)	145 (14.9)	399 (14.9)	869 (11.3)
Very poor, n (%)	48 (1.2)	30 (3.1)	72 (2.7)	150 (1.9)
**ACT score^ƒ^**				
Patients with data, n	3986	877	NA	4863
Mean±sd	19.5±4.6	17.7±5.1	NA	19.2±4.8
**RSQ score^##^**				
Patients with data, n	4119	985	2693	7797
Mean±sd	4.6±4.0	6.3±4.4	5.8±4.1	5.2±4.1
**Physician-reported exacerbations in the past 12 months**				
Patients with data, n	4125	989	2690	7804
Mean±sd	0.57±1.1	0.93±1.4	0.57±1.0	0.62±1.1

This table includes data for all NOVELTY patients with a baseline Chronic Airways Assessment Test (CAAT) and no missing baseline data for physician-assigned diagnosis and physician-assessed severity. Overall health status was assessed from the introductory question to the St George's Respiratory Questionnaire (SQRQ), “Please tick in one box to show how you describe your current health”. ACT: Asthma Control Test; FEV_1_: forced expiratory volume in 1 s; FVC: forced vital capacity; mMRC: modified Medical Research Council; N: total number of patients in the group; n: number of patients with non-missing data; NA: not applicable; RSQ: Respiratory Symptoms Questionnaire. ^#^: For patients with asthma+COPD, severity was allocated as the higher of the two severity categories assigned by the physician for their asthma and their COPD. ^¶^: Range: 0–40. ^+^: Range: 0–4. ^§^: Range: 0–100. ^ƒ^: Range: 5–25. ^##^: Range: 0–16.

Mean±sd CAAT score in all patients was 15.4±8.6 and was lower in patients with asthma (14.0±8.5) compared with patients with asthma+COPD (17.2±8.6) or COPD (17.0±8.3) (table 1). The majority (71.3%) of patients had a CAAT score of ≥10; cut-off values for the CAAT have not yet been determined, but in COPD, a CAT score of ≥10 indicates medium-to-very high impact on patients’ daily lives [29], although the impact level based on CAAT score has not yet been determined. There were almost no patients who showed a ceiling effect, even in severe asthma and COPD. Floor effects were seen in <3% of mild asthma and even fewer in mild asthma+COPD and mild COPD. Comorbidities were common, with 53.2% of patients having at least one respiratory comorbidity and 66.4% having at least one nonrespiratory comorbidity (supplementary table 2).

### Association between the CAAT and clinical characteristics

**FIGURE 1:**
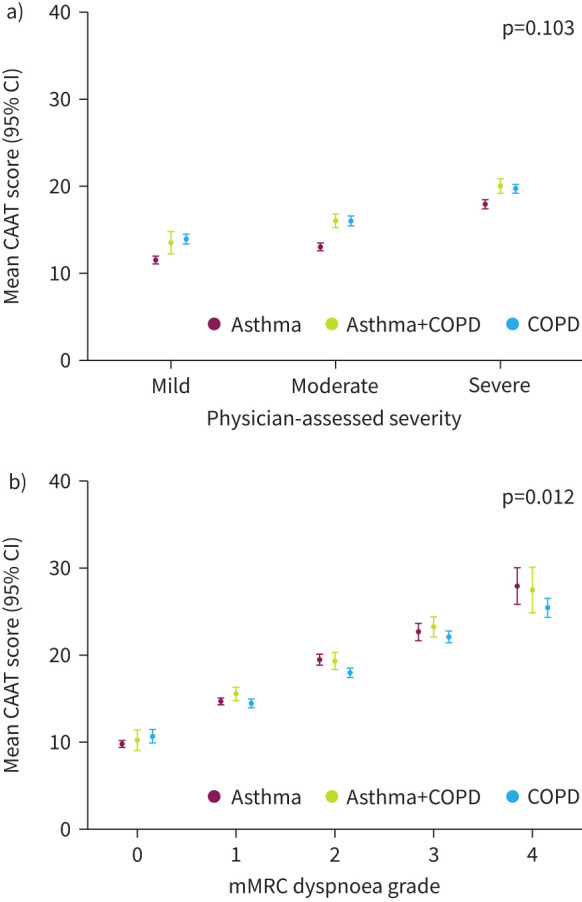
Association of Chronic Airways Assessment Test (CAAT) score with categorical variables. a) Physician-assessed severity and b) modified Medical Research Council (mMRC) dyspnoea grade in patients with asthma, asthma+COPD or COPD. The figure shows interaction plots designed to visualise any differences in the association between CAAT score and each clinical characteristic between diagnostic groups. To test whether the association between CAAT score and each clinical characteristic differed between diagnostic groups, an interaction term was included in each model. Physician-assessed severity and mMRC dyspnoea grade were analysed as categorical variables.

**FIGURE 2:**
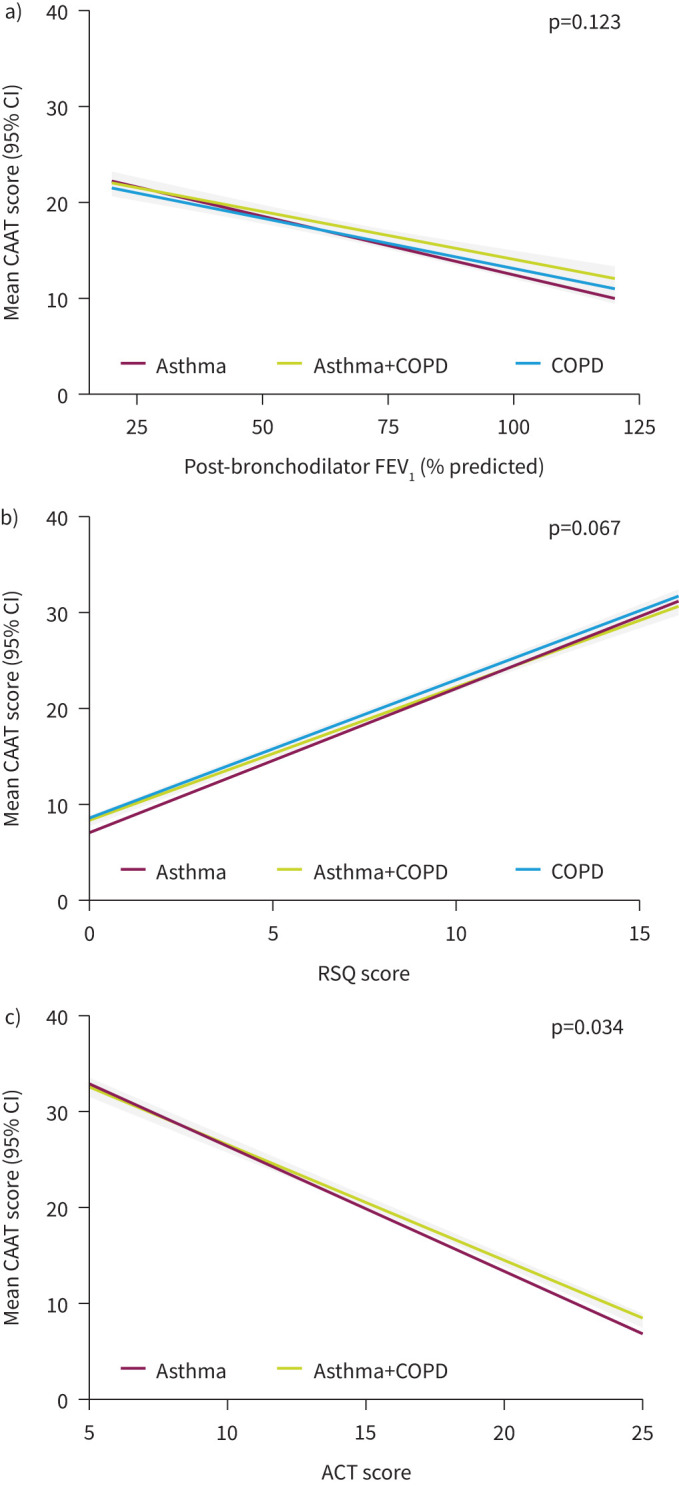
Association of Chronic Airways Assessment Test (CAAT) score with continuous variables. a) Post-bronchodilator forced expiratory volume in 1 s (FEV_1_ % pred) and b) respiratory symptom score (RSQ) in patients with asthma, asthma+COPD or COPD and c) Asthma Control Test (ACT) score in patients with asthma or asthma+COPD. The figure shows interaction plots designed to visualise any differences in the association between CAAT score and each clinical characteristic between diagnostic groups. To test whether the association between CAAT score and each clinical characteristic differed between diagnostic groups, an interaction term was included in each model. Post-bronchodilator FEV_1_% pred, RSQ score and ACT score were analysed as continuous variables. Grey bands indicate 95% confidence intervals.

Results from the unadjusted linear regression analyses are shown in table 2 and in figures 1 and 2 and estimated marginal trends and contrasts in supplementary tables 3 and 4. Higher CAAT scores were associated with worse levels of all clinical characteristics across all three diagnostic groups; CAAT scores were higher with worse physician-assigned severity (figure 1a), higher mMRC dyspnoea grade (figure 1b), lower post-bronchodilator FEV_1_% pred (figure 2a), higher RSQ score (figure 2b) and lower ACT score (figure 2c). The associations between CAAT score and each of these clinical characteristics showed similar trends across diagnostic groups (table 2, figure 1 and figure 2). For physician-assessed severity, mean CAAT scores were consistently lower (by ≥2 points in most cases) in asthma compared with asthma+COPD or COPD at each severity level (figure 1a). The unadjusted p-values for the interaction tests for mMRC dyspnoea grade and ACT score were 0.012 and 0.034, respectively. The unadjusted p-values for the interaction tests for all other clinical characteristics were >0.05.

**TABLE 2:** Association between Chronic Airways Assessment Test (CAAT) and clinical features from regression models, with interaction term for diagnosis

Model	Parameter estimate	95% CI	Type 3 p-value for interaction by diagnosis
**Physician-assessed severity**			0.103
Asthma+COPD *versus* asthma	2.00	0.71–3.29	
COPD *versus* asthma	2.41	1.71–3.12	
Moderate *versus* mild	1.49	0.92–2.07	
Severe *versus* mild	6.42	5.81–7.03	
**mMRC dyspnoea grade**			0.012
Asthma+COPD *versus* asthma	0.42	−0.81–1.65	
COPD *versus* asthma	0.84	0.00–1.69	
Grade 1 *versus* grade 0	4.88	4.37–5.39	
Grade 2 *versus* grade 0	9.64	8.93–10.35	
Grade 3 *versus* grade 0	12.86	11.79–13.92	
Grade 4 *versus* grade 0	18.12	16.02–20.22	
**Lung function (post-bronchodilator FEV_1_, % pred)**			0.123
Asthma+COPD *versus* asthma	−0.66	−2.82–1.50	
COPD *versus* asthma	−1.16	−2.67–0.35	
FEV_1_ % pred	−0.12	−0.14– −0.11	
**RSQ**			0.067
Asthma+COPD *versus* asthma	1.30	0.59–2.01	
COPD *versus* asthma	1.51	1.04–1.99	
RSQ	1.50	1.45–1.55	
**ACT**			0.034
Asthma+COPD *versus* asthma	−0.71	−2.34–0.92	
ACT	−1.30	−1.34– −1.26	

The presented values are the main effect estimates and 95% confidence intervals from the linear regression models shown in figures 1 and 2. The Respiratory Symptoms Questionnaire (RSQ) has been validated in the NOVELTY population previously [28]. ACT: Asthma Control Test; FEV_1_: forced expiratory volume in 1 s; mMRC: modified Medical Research Council.

Similar findings were observed when the linear regression analyses were adjusted for age (supplementary figures 4 and 5) and age, sex and smoking status (supplementary figures 6 and 7).

## Discussion

The CAAT demonstrated cross-sectional validity across asthma, asthma+COPD and COPD, suggesting that it is an acceptable measure of health status in patients with any of these diagnoses. As expected, higher CAAT scores were associated with worse levels of several clinical characteristics across all three diagnostic groups. This builds upon results from the previous psychometric analysis [19], in which higher CAAT scores were associated with worse SGRQ and EQ-5D-5L VAS scores, and strengthens the evidence for the suitability of the CAAT as a short, simple health status measure for routine use in research and clinical practice across respiratory diseases.

In all three diagnostic groups, higher CAAT scores were associated with higher respiratory symptom frequency and impact assessed by the RSQ, worse activity limitation due to breathlessness as assessed by mMRC grade and, in patients with a diagnosis of asthma with or without COPD, worse asthma symptom control as indicated by lower ACT score. All these variables reflect the substantial burden experienced by patients with chronic or recurrent respiratory symptoms and their impact on patients’ health status [30, 31]. The relatively flat association between post-bronchodilator FEV_1_% pred and CAAT score in all three diagnostic groups is consistent with previous observations for CAT in COPD [32], indicating that lung function does not reflect the full impact of chronic respiratory disease on patients and emphasising the importance of a more comprehensive patient-centred evaluation.

We observed that CAAT scores were consistently lower in patients with asthma compared with those with asthma+COPD or COPD across physician-assessed severities (by ≥2 points in most between-group comparisons). Although the mean age of patients with asthma was lower than those with COPD and those with asthma+COPD, adjusting the analyses for age did not affect the results and the differences also remained after adjustment for age, sex and smoking status. These differences are not surprising, as in each of the three categories of physician-assessed severity (mild, moderate, severe) previously, patients with a diagnosis of asthma had higher lung function, lower mMRC grades (less shortness of breath) and better overall health status than those with asthma+COPD or COPD; the differences between asthma+COPD and COPD for each of these variables were smaller [20]. Indeed, in this analysis, there was a large difference in group mean lung function between the asthma and COPD groups (mean post-bronchodilator FEV_1_% pred was 86.9% for asthma *versus* 61% in COPD). However, for patients with similar mean values for FEV_1_% pred, there were only small differences in CAAT score between the diagnostic groups (figure 2a). There may also have been differences in the way that physicians assessed severity in asthma and COPD in NOVELTY; they were deliberately not given any instructions about how to assess severity and there is no standard concept of severity across the spectrum of asthma and/or COPD. A previous NOVELTY study identified several clinical and spirometric factors that were significantly associated with physician-assigned severity in asthma and/or COPD [20]. However, adjusting the present analysis for severity could have hindered the ability to observe differences in associations between the CAAT scores and clinical characteristics by diagnosis. Therefore, we adjusted for age, gender and smoking status, which together should explain a relatively large degree of variability.

In the linear regression analyses testing the association between CAAT score and mMRC grade, and CAAT score and ACT score (figures 1b and 2c, respectively) the interaction term to test whether the slopes were different between groups had p-values >0.01 but <0.05. Inspection of the plots showed that the slopes were very similar and any differences in CAAT scores were small. Overall, the similar relationship between CAAT score and the other clinical measures captured in this study, as illustrated by figures 1 and 2, highlight the validity of the CAAT across asthma, asthma+COPD and COPD. In addition, the CAAT score was normally distributed with no strong boundary effects, which suggests this tool is sensitive enough to detect differences at the extreme ends of the scale.

While asthma and COPD are distinct diseases with differences in aetiology and prognosis, there are similarities in some pathophysiological characteristics between asthma and COPD [33] and in the development of persistent airflow limitation in patients with long-standing asthma [34]. It is therefore particularly important for clinicians and researchers to have access to convenient and practical diagnosis-agnostic PROs that can be used to assess health status across the whole spectrum of airways disease, including those patients with diagnoses of both asthma and COPD, as well as those who do not yet have a confirmed diagnosis. The CAAT includes items such as mucus production, lack of confidence and lack of energy that are often found in patients with asthma [4, 35, 36], but these are not assessed in current asthma symptom control tools such as the ACT and the ACQ [10, 11].

The strengths and limitations of this analysis are largely those of the NOVELTY study overall, which have been reported previously [20, 23]. A particular strength of this study is that the analysis was performed in a large, diverse, real-world population of patients with asthma and/or COPD at different levels of physician-assessed severity from 18 countries. From the limitations perspective, the NOVELTY population is not a random sample, as recruitment was stratified in each country or region with target numbers by diagnosis and severity to ensure sufficient sub-group samples for analysis. Future longitudinal analyses are needed to assess the performance of the CAAT over time and in other airways diseases, including bronchiectasis, and to validate the minimum clinically important difference for the CAAT in these conditions. In addition, further work to examine the effect of respiratory comorbidities and systemic comorbidities on the CAAT may be important for certain patients; for example, rhinitis may have an impact for those with asthma and systemic comorbidities for older patients with asthma and/or COPD. Defining disease-specific CAAT score thresholds that identify patients with poor or good disease control will also be clinically useful. Our analyses suggest that an ACT score of 20, an accepted criterion of good control in asthma [37], corresponds to a CAAT score of ∼12–13. Furthermore, a CAAT score of 10 (which for CAT in COPD is a well-established threshold for disease control [29]), corresponds to an ACT score of approximately 23, which also indicates good control in asthma.

In conclusion, this analysis builds upon the findings of the previous psychometric analysis [19] demonstrating that the CAAT has consistent cross-sectional validity across asthma, asthma+COPD and COPD, making it suitable for assessment of health status in adults in research and in clinical practice. The CAAT includes items that are of importance to patients and are relevant to both asthma and COPD. It is also practical and convenient for use in routine clinical practice. Its use may also support research around the impact of lung disease in populations where the diagnosis of asthma or COPD is unclear or has not been confirmed.

## Data Availability

Data underlying the findings described in this manuscript may be obtained in accordance with AstraZeneca's data sharing policy described at https://astrazenecagrouptrials.pharmacm.com/ST/Submission/Disclosure. Data for studies directly listed on Vivli can be requested through Vivli at www.vivli.org. Data for studies not listed on Vivli could be requested through Vivli at https://vivli.org/members/enquiries-about-studies-not-listed-on-the-vivli-platform/. AstraZeneca Vivli member page is also available outlining further details: https://vivli.org/ourmember/astrazeneca/. The NOVELTY protocol is available at https://astrazenecagrouptrials.pharmacm.com.

## Supplementary material

**Please note:** supplementary material is not edited by the Editorial Office, and is uploaded as it has been supplied by the author. Supplementary material 01359-2024.SUPPLEMENT
