# Supplementary material for: Validity of the Chronic Airways Assessment Test (CAAT) in asthma, asthma+COPD and COPD in NOVELTY
Source: ERJ Open Res. 2025 Jul 21;11(4):01359-2024. doi: 10.1183/23120541.01359-2024 (PMC12278303; doi:10.1183/23120541.01359-2024)
Supplement: Supplementary file 1 [file 01359-2024.SUPPLEMENT.pdf]

SUPPLEMENTARY MATERIAL

SUPPLEMENTARY TABLE 1 Patient baseline clinical outcome score groups by physician-assigned diagnostic group

	**ASTHMA (N=4138)**	**ASTHMA+ COPD (N=991)**	**COPD (N=2699)**	**Total (N=7828)**
CAAT score group[a]				
Patients with data, n	4138	991	2699	7828
<10, n (%)	1464 (35.4)	207 (20.9)	574 (21.3)	2245 (28.7)
≥10 to ≤20	1733 (41.9)	428 (43.2)	1194 (44.2)	3355 (42.9)
>20 to ≤30	787 (19.0)	288 (29.1)	780 (28.9)	1855 (23.7)
>30	154 (3.7)	68 (6.9)	151 (5.6)	373 (4.8)
mMRC dyspnoea grade				
Patients with data, n	3982	957	2636	7575
0	1586 (39.8)	149 (15.6)	351 (13.3)	2086 (27.5)
1	1593 (40.0)	401 (41.9)	918 (34.8)	2912 (38.4)
2	551 (13.8)	223 (23.3)	709 (26.9)	1483 (19.6)
3	204 (5.1)	154 (16.1)	486 (18.4)	844 (11.1)
4	48 (1.2)	30 (3.1)	172 (6.5)	250 (3.3)
ACT score group[b,c]				
Patients with data, n	3986	877	NA	4863
Well-controlled, n (%)	2359 (59.2)	364 (41.5)	NA	2723 (56.0)
Not well-controlled, n (%)	818 (20.5)	229 (26.1)	NA	1047 (21.5)
Very poorly controlled, n (%)	809 (20.3)	284 (32.4)	NA	1093 (22.5)

[a]Range: 0–40. In patients with COPD, CAT score of <10 indicates low respiratory health impact [1]. [b]Range: 5–25. [c]Very poorly controlled was defined as ACT score ≤15, not well-controlled was defined as ACT score 16–19, and well-controlled was defined as ACT score ≥20 [2].

ACT: Asthma Control Test; CAAT: Chronic Airways Assessment Test; COPD: chronic obstructive pulmonary disease; mMRC: modified Medical Research Council; N: total number of patients in the group; n: number of patients with non-missing data; NA: not applicable.

SUPPLEMENTARY TABLE 2 Patient baseline respiratory and non-respiratory comorbidities by physician-assigned diagnosis group

	**ASTHMA (N=4138)**	**ASTHMA+ COPD (N=991)**	**COPD (N=2699)**	**Total (N=7828)**
Respiratory comorbidities, n (%)				
Patients with data, n	4138	991	2699	7828
Any respiratory comorbidity	2070 (50.0)	634 (64.0)	1457 (54.0)	4161 (53.2)
Allergic rhinitis	1308 (31.6)	241 (24.3)	156 (5.8)	1705 (21.8)
Obstructive sleep apnoea	278 (6.7)	101 (10.2)	245 (9.1)	624 (8.0)
Recurrent/chronic non-allergic rhinitis/sinusitis	411 (9.9)	92 (9.3)	80 (3.0)	583 (7.4)
Chronic bronchitis	93 (2.2)	76 (7.7)	151 (5.6)	320 (4.1)
Bronchiectasis	134 (3.2)	50 (5.0)	98 (3.6)	282 (3.6)
Nasal/sinus polyps	233 (5.6)	34 (3.4)	14 (0.5)	281 (3.6)
Lung cancer	4 (0.1)	6 (0.6)	34 (1.3)	44 (0.6)
Evidence of past pulmonary tuberculosis	13 (0.3)	8 (0.8)	36 (1.3)	57 (0.7)
Vocal cord dysfunction	17 (0.4)	9 (0.9)	10 (0.4)	36 (0.5)
Sarcoidosis	15 (0.4)	8 (0.8)	11 (0.4)	34 (0.4)
Dysfunctional breathing	16 (0.4)	6 (0.6)	5 (0.2)	27 (0.3)
Idiopathic pulmonary fibrosis	5 (0.1)	4 (0.4)	7 (0.3)	16 (0.2)
Pneumoconiosis	2 (0.0)	0 (0.0)	8 (0.3)	10 (0.1)
Silicosis	1 (0.0)	0 (0.0)	2 (0.1)	3 (0.0)
Active pulmonary tuberculosis	1 (0.0)	0 (0.0)	0 (0.0)	1 (0.0)
Non-respiratory comorbidities, n (%)				
Patients with data, n	4138	991	2699	7828
Any non-respiratory comorbidity	2309 (55.8)	783 (79.0)	2104 (78.0)	5196 (66.4)
Type I diabetes	26 (0.6)	5 (0.5)	8 (0.3)	39 (0.5)
Type II diabetes	387 (9.4)	147 (14.8)	428 (15.9)	962 (12.3)
Retinopathy related to diabetes	8 (0.2)	3 (0.3)	8 (0.3)	19 (0.2)
Nephropathy related to diabetes	9 (0.2)	5 (0.5)	9 (0.3)	23 (0.3)

Neuropathy related to diabetes	7 (0.2)	7 (0.7)	13 (0.5)	27 (0.3)
Hypercholesterolaemia	540 (13.0)	212 (21.4)	634 (23.5)	1386 (17.7)
Previous stroke	37 (0.9)	24 (2.4)	54 (2.0)	115 (1.5)
Transient ischaemic attack	27 (0.7)	21 (2.1)	43 (1.6)	91 (1.2)
Peripheral vascular disease	13 (0.3)	27 (2.7)	111 (4.1)	151 (1.9)
Coronary artery disease	81 (2.0)	77 (7.8)	251 (9.3)	409 (5.2)
Hypertension	1079 (26.1)	385 (38.8)	1226 (45.4)	2690 (34.4)
Myocardial infarction	49 (1.2)	35 (3.5)	130 (4.8)	214 (2.7)
Cardiac arrhythmia	135 (3.3)	57 (5.8)	169 (6.3)	361 (4.6)
Congestive heart failure	23 (0.6)	20 (2.0)	112 (4.1)	155 (2.0)
Anxiety	267 (6.5)	104 (10.5)	222 (8.2)	593 (7.6)
Depression	367 (8.9)	137 (13.8)	276 (10.2)	780 (10.0)
Other psychiatric disorders	67 (1.6)	13 (1.3)	40 (1.5)	120 (1.5)
Osteoarthritis or unspecified arthritis	347 (8.4)	158 (15.9)	303 (11.2)	808 (10.3)
Rheumatoid arthritis	67 (1.6)	23 (2.3)	47 (1.7)	137 (1.8)
Osteoporosis	205 (5.0)	73 (7.4)	170 (6.3)	448 (5.7)
Inflammatory bowel disease	50 (1.2)	20 (2.0)	28 (1.0)	98 (1.3)
Thyroid disease	340 (8.2)	85 (8.6)	227 (8.4)	652 (8.3)
Chronic kidney disease	36 (0.9)	33 (3.3)	64 (2.4)	133 (1.7)
Gastroesophageal reflux disease	618 (14.9)	202 (20.4)	384 (14.2)	1204 (15.4)
HIV/AIDS	5 (0.1)	4 (0.4)	11 (0.4)	20 (0.3)
Malignancy in complete remission	108 (2.6)	53 (5.3)	140 (5.2)	301 (3.8)
Active malignancy	29 (0.7)	15 (1.5)	54 (2.0)	98 (1.3)

AIDS: acquired immunodeficiency syndrome; HIV: human immunodeficiency virus; N: total number of patients in the group; n: number of patients with non-missing data.

SUPPLEMENTARY TABLE 3 Mean CAAT difference between severity levels and mMRC grades in disease diagnosis groups

Pairwise comparisons by physician-assigned diagnostic group	Contrast	SE	p-value
**Severity**			
Overall			
Moderate-Mild	2.02	0.30	<0.001
Severe-Mild	6.23	0.30	<0.001
Asthma			
Moderate-Mild	1.49	0.30	<0.001
Severe-Mild	6.42	0.31	<0.001
Asthma+COPD			
Moderate-Mild	2.52	0.73	0.01
Severe-Mild	6.51	0.75	<0.001
COPD			
Moderate-Mild	2.05	0.40	<0.001
Severe-Mild	5.75	0.38	<0.001
**mMRC grade**			
Overall			
Grade 1–Grade 0	4.66	0.29	<0.001
Grade 2–Grade 0	8.67	0.33	<0.001
Grade 3–Grade 0	12.43	0.37	<0.001
Grade 4–Grade 0	16.72	0.65	<0.001
Asthma			
Grade 1–Grade 0	4.88	0.26	<0.001
Grade 2–Grade 0	9.63	0.36	<0.001
Grade 3–Grade 0	12.85	0.54	<0.001
Grade 4–Grade 0	18.12	1.07	<0.001
Asthma+COPD			
Grade 1–Grade 0	5.33	0.70	<0.001

Grade 2–Grade 0	9.09	0.77	<0.001
Grade 3–Grade 0	13.0	0.84	<0.001
Grade 4–Grade 0	17.25	1.46	<0.001
COPD			
Grade 1–Grade 0	3.78	0.46	<0.001
Grade 2–Grade 0	7.30	0.48	<0.001
Grade 3–Grade 0	11.42	0.51	<0.001
Grade 4–Grade 0	14.80	0.68	<0.001

Pairwise comparisons are described by contrasts and SE.

CAAT, Chronic Airways Assessment Test; COPD, chronic obstructive pulmonary disease; mMRC, modified Medical Research Council; SE, standard error.

SUPPLEMENTARY TABLE 4 CAAT change per unit increase in FEV_1 (% predicted), ACT and RSQ score

Variable by physician-assigned diagnostic group	Marginal trend	SE	95% CI
**FEV_1 (% predicted)**			
Overall	−0.11	0.01	−0.12; −0.10
Asthma	−0.12	0.01	−0.14; −0.11
Asthma+COPD	−0.10	0.01	−0.13; −0.08
COPD	−0.11	0.01	−0.12; −0.09
**ACT**			
Overall	−1.25	0.02	−1.29; −1.21
Asthma	−1.30	0.02	−1.34; −1.26
Asthma+COPD	−1.20	0.04	−1.28; −1.13
**RSQ**			
Overall	1.45	0.02	1.41; 1.48
Asthma	1.50	0.02	1.45; 1.55
Asthma+COPD	1.39	0.04	1.31; 1.48
COPD	1.45	0.03	1.39; 1.50

ACT, Asthma Control Test; CAAT, Chronic Airways Assessment Test; CI, confidence interval; COPD, chronic obstructive pulmonary disease; FEV_1 (% predicted), forced expiratory volume in 1 second as a percentage of the predicted normal value; RSQ, Respiratory Symptoms Questionnaire; SE, standard error.

SUPPLEMENTARY TABLE 5 List of NOVELTY Scientific Community members

**Scientific Community member**	**Country**	**Scientific Community member**	**Country**
Ricardo del Olmo	Argentina	Stefan Franzén	AstraZeneca, Sweden
Gary Anderson	Australia	Christina Keen	AstraZeneca, Sweden
Helen Reddel	Australia	Kristoffer Ostridge	AstraZeneca, Sweden
Marcelo Rabahi	Brazil	James Chalmers	UK
Andrew McIvor	Canada	Timothy Harrison	UK
Mohsen Sadatsafavi	Canada	Ian Pavord	UK
Ulla Weinreich	Denmark	David Price	UK
Pierre-Régis Burgel	France	Adnan Azim	AstraZeneca, UK
Gilles Devouassoux	France	Laura Belton	AstraZeneca, UK
Alberto Papi	Italy	Francois-Xavier Blé	AstraZeneca, UK
Hiromasa Inoue	Japan	Clement Erhard	AstraZeneca, UK
Adrián Rendon	Mexico	Rod Hughes	AstraZeneca, UK
Maarten van den Berge	Netherlands	Glenda Lassi	AstraZeneca, UK
Richard Beasley	New Zealand	Hana Müllerová	AstraZeneca, UK
Alvar Agusti García-Navarro	Spain	Deven Patel	AstraZeneca, UK
Rosa Faner	Spain	Eleni Rapsomaniki	AstraZeneca, UK
José Olaguibel Rivera	Spain	Ian Christopher Scott	AstraZeneca, UK
Christer Janson	Sweden	Bradley Chipps	USA
Magdalena Bilińska-Izydorczyk	AstraZeneca, Sweden	Stephanie Christenson	USA
Malin Fagerås	AstraZeneca, Sweden	Barry Make	USA
Titti Fihn-Wikander	AstraZeneca, Sweden	Erin Tomaszewski	AstraZeneca, USA

SUPPLEMENTARY TABLE 6 List of NOVELTY study investigators

Investigator	Country	Investigator	Country
**Ricardo del Olmo**	Argentina	**Hiromasa Inoue**	Japan
Gabriel Benhabib	Argentina	Takeo Endo	Japan
Xavier Bocca Ruiz	Argentina	Masaki Fujita	Japan
Raul Eduardo Lisanti	Argentina	Yu Hara	Japan
Gustavo Marino	Argentina	Takahiko Horiguchi	Japan
Walter Mattarucco	Argentina	Keita Hosoi	Japan
Juan Nogueira	Argentina	Yumiko Ide	Japan
Maria Parody	Argentina	Minehiko Inomata	Japan
Pablo Pascale	Argentina	Koji Inoue	Japan
Pablo Rodriguez	Argentina	Sumito Inoue	Japan
Damian Silva	Argentina	Motokazu Kato	Japan
Graciela Svetliza	Argentina	Masayuki Kawasaki	Japan
Carlos F. Victorio	Argentina	Tomotaka Kawayama	Japan
Roxana Willigs Rolon	Argentina	Toshiyuki Kita	Japan
Anahi Yañez	Argentina	Kanako Kobayashi	Japan
**Helen Reddel**	Australia	Hiroshi Koto	Japan
Stuart Baines	Australia	Koichi Nishi	Japan
Simon Bowler	Australia	Junpei Saito	Japan
Peter Bremner	Australia	Yasuo Shimizu	Japan
Sheetal Bull	Australia	Toshihiro Shirai	Japan
Patrick Carroll	Australia	Naruhiko Sugihara	Japan
Mariam Chaalan	Australia	Ken-ichi Takahashi	Japan
Claude Farah	Australia	Hiroyuki Tashimo	Japan
Gary Hammerschlag	Australia	Keisuke Tomii	Japan
Kerry Hancock	Australia	Takashi Yamada	Japan
Zinta Harrington	Australia	Masaru Yanai	Japan
Gregory Katsoulotos	Australia	**Adrián Rendon**	Mexico
Joshua Kim	Australia	Ruth Cerino Javier	Mexico
David Langton	Australia	Alfredo Domínguez Peregrina	Mexico
Donald Lee	Australia	Marco Fernández Corzo	Mexico
Matthew Peters	Australia	Efraín Montano Gonzalez	Mexico
Lakshman Prassad	Australia	Alejandra Ramírez-Venegas	Mexico

Dimitar Sajkov	Australia	**Maarten van den Berge**	Netherlands
Francis Santiago	Australia	Willem Boersma	Netherlands
Frederick Graham Simpson	Australia	R.S. Djamin	Netherlands
Sze Tai	Australia	Michiel Eijsvogel	Netherlands
Paul Thomas	Australia	Frits Franssen	Netherlands
Peter Wark	Australia	Martijn Goosens	Netherlands
**Marcelo Rabahi**	Brazil	Lidwien Graat-Verboom	Netherlands
José Eduardo Delfini Cançado	Brazil	Johannes in 't Veen	Netherlands
Thúlio Cunha	Brazil	Rob Janssen	Netherlands
Marina Lima	Brazil	Kim Kuppens	Netherlands
Alexandre Pinto Cardoso	Brazil	Mario van de Ven	Netherlands
**J. Mark FitzGerald**	Canada	**Per Bakke**	Norway
**Andrew McIvor**	Canada	Ole Petter Brunstad	Norway
Syed Anees	Canada	Gunnar Einvik	Norway
John Bertley	Canada	Kristian Jong Høines	Norway
Alan Bell	Canada	Alamdar Khusrawi	Norway
Amarjit Cheema	Canada	Torbjorn Oien	Norway
Guy Chouinard	Canada	**Ho Joo Yoon**	South Korea
Michael Csanadi	Canada	Yoon-Seok Chang	South Korea
Anil Dhar	Canada	Young Joo Cho	South Korea
Ripple Dhillon	Canada	Yong Il Hwang	South Korea
David Kanawaty	Canada	Woo Jin Kim	South Korea
Allan Kelly	Canada	Young-Il Koh	South Korea
William Killorn	Canada	Byung-Jae Lee	South Korea
Daniel Landry	Canada	Kwan-Ho Lee	South Korea
Robert Luton	Canada	Sang-Pyo Lee	South Korea
Piushkumar Mandhane	Canada	Yong Chul Lee	South Korea
Bonavuth Pek	Canada	Seong Yong Lim	South Korea
Robert Petrella	Canada	Kyung Hun Min	South Korea
Daniel Stollery	Canada	Yeon-Mok Oh	South Korea
**Chen Wang**	China[a]	Choon-Sik Park	South Korea
Meihua Chen	China[a]	Hae-Sim Park	South Korea
Yan Chen	China[a]	Heung-Woo Park	South Korea
Wei Gu	China[a]	Chin Kook Rhee	South Korea
Kim Ming Christopher Hui	China[a]	Hyoung-Kyu Yoon	South Korea

Manxiang Li	China[a]	**Alvar Agusti García-Navarro**	Spain
Shiyue Li	China[a]	**José Olaguibel Rivera**	Spain
Ma Lijun	China[a]	Rubén Andújar	Spain
Guangyue Qin	China[a]	Laura Anoro	Spain
Weidong Song	China[a]	María Buendía García	Spain
Wei Tan	China[a]	Paloma Campo Mozo	Spain
Yijun Tang	China[a]	Sergio Campos	Spain
Tan Wang	China[a]	Francisco Casas Maldonado	Spain
Fuqiang Wen	China[a]	Manuel Castilla Martínez	Spain
Feng Wu	China[a]	Carolina Cisneros Serrano	Spain
PingChao Xiang	China[a]	Lorena Comeche Casanova	Spain
Zuke Xiao	China[a]	Dolores Corbacho	Spain
Shengdao Xiong	China[a]	Felix Del Campo Matías	Spain
Jinghua Yang	China[a]	Jose Echave-Sustaeta	Spain
Jingping Yang	China[a]	Gloria Francisco Corral	Spain
Caiqing Zhang	China[a]	Pedro Gamboa Setién	Spain
Min Zhang	China[a]	Marta García Clemente	Spain
Ping Zhang	China[a]	Ignacio García Núñez	Spain
Wei Zhang	China[a]	Jose García Robaina	Spain
Xiaohe Zheng	China[a]	Mercedes García Salmones	Spain
Dan Zhu	China[a]	Jose Maria Marín Trigo	Spain
**Carlos Matiz Bueno**	Colombia	Marta Nuñez Fernandez	Spain
Fabio Bolivar Grimaldos	Colombia	Sara Nuñez Palomo	Spain
Alejandra Cañas Arboleda	Colombia	Luis Pérez de Llano	Spain
Dora Molina de Salazar	Colombia	Ana Pueyo Bastida	Spain
**Ulla Weinreich**	Denmark	Ana Rañó	Spain
Elisabeth Bendstrup	Denmark	José Rodríguez González-Moro	Spain
Ole Hilberg	Denmark	Albert Roger Reig	Spain
Carsten Kjellerup	Denmark	José Velasco Garrido	Spain
**Pierre-Régis Burgel**	France	**Christer Janson**	Sweden
**Gilles Devouassoux**	France	Dan Curiac	Sweden
**Chantal Raherison**	France	Cornelia Lif-Tiberg	Sweden
Philippe Bonniaud	France	Anders Luts	Sweden

Olivier Brun	France	Lennart Råhlen	Sweden
Christos Chouaid	France	Stefan Rustscheff	Sweden
Francis Couturaud	France	**Timothy Harrison**	UK
Jacques de Blic	France	Frances Adams	UK
Didier Debieuvre	France	Drew Bradman	UK
Dominique Delsart	France	Emma Broughton	UK
Axelle Demaegdt	France	John Cosgrove	UK
Pascal Demoly	France	Patrick Flood-Page	UK
Antoine Deschildre	France	Elizabeth Fuller	UK
Carole Egron	France	David Hartley	UK
Lionel Falchero	France	Keith Hattotuwa	UK
François Goupil	France	Gareth Jones	UK
Romain Kessler	France	Keir Lewis	UK
Pascal Le Roux	France	Lorcan McGarvey	UK
Pascal Mabire	France	Alyn Morice	UK
Guillaume Mahay	France	Preeti Pandya	UK
Stéphanie Martinez	France	Manish Patel	UK
Boris Melloni	France	Kay Roy	UK
Laurent Moreau	France	Ramamurthy Sathyamurthy	UK
Emilie Riviere	France	Swaminathan Thiagarajan	UK
Pauline Roux-Claudé	France	Alice Turner	UK
Michel Soulier	France	Jorgen Vestbo	UK
Guillaume Vignal	France	Wisia Wedzicha	UK
Azzedine Yaici	France	Tom Wilkinson	UK
**Robert Bals**	Germany	Pete Wilson	UK
Sven Philip Aries	Germany	**Bradley Chipps**	USA
Ekkehard Beck	Germany	Lo'Ay Al-Asadi	USA
Andreas Deimling	Germany	James Anholm	USA
Jan Feimer	Germany	Francis Averill	USA
Vera Grimm-Sachs	Germany	Sandeep Bansal	USA
Gesine Groth	Germany	Alan Baptist	USA
Felix Herth	Germany	Colin Campbell	USA
Gerhard Hoheisel	Germany	Michael A. Campos	USA
Frank Kanniess	Germany	Gretchen Crook	USA
Thomas Lienert	Germany	Samuel DeLeon	USA

Silke Mronga	Germany	Alain Eid	USA
Jörg Reinhardt	Germany	Ellen Epstein	USA
Christian Schlenska	Germany	Stephen Fritz	USA
Christoph Stolpe	Germany	Hoadley Harris	USA
Ishak Teber	Germany	Mitzie Hewitt	USA
Hartmut Timmermann	Germany	Fernando Holguin	USA
Thomas Ulrich	Germany	Golda Hudes	USA
Peter Velling	Germany	Richard Jackson	USA
Sabina Wehgartner-Winkler	Germany	Alan Kaufman	USA
Juergen Welling	Germany	David Kaufman	USA
Ernst-Joachim Winkelmann	Germany	Ari Klapholz	USA
**Alberto Papi**	Italy	Harshavardhan Krishna	USA
Carlo Barbetta	Italy	Daria Lee	USA
Fulvio Braido	Italy	Robert Lin	USA
Vittorio Cardaci	Italy	Diego Maselli-Caceres	USA
Enrico Maria Clini	Italy	Vinay Mehta	USA
Maria Teresa Costantino	Italy	James N. Moy	USA
Giuseppina Cuttitta	Italy	Ugo Nwokoro	USA
Mario di Gioacchino	Italy	Purvi Parikh	USA
Alessandro Fois	Italy	Sudhir Parikh	USA
Maria Pia Foschino-Barbaro	Italy	Frank Perrino	USA
Enrico Gammeri	Italy	James Ruhlmann	USA
Riccardo Inchingolo	Italy	Catherine Sassoon	USA
Federico Lavorini	Italy	Russell A. Settipane	USA
Antonio Molino	Italy	Daniel Sousa	USA
Eleonora Nucera	Italy	Peruvemba Sriram	USA
Vincenzo Patella	Italy	Richard Wachs	USA
Alberto Pesci	Italy		
Fabio Ricciardolo	Italy		
Paola Rogliani	Italy		
Riccardo Sarzani	Italy		
Carlo Vancheri	Italy		
Rigoletta Vincenti	Italy		

National Principal Investigators are shown in bold. [a]Data for patients from China were excluded from the present analyses due to a change in regulations about data transfer in May 2019.

SUPPLEMENTARY FIGURE 1 The CAAT, response options and scoring

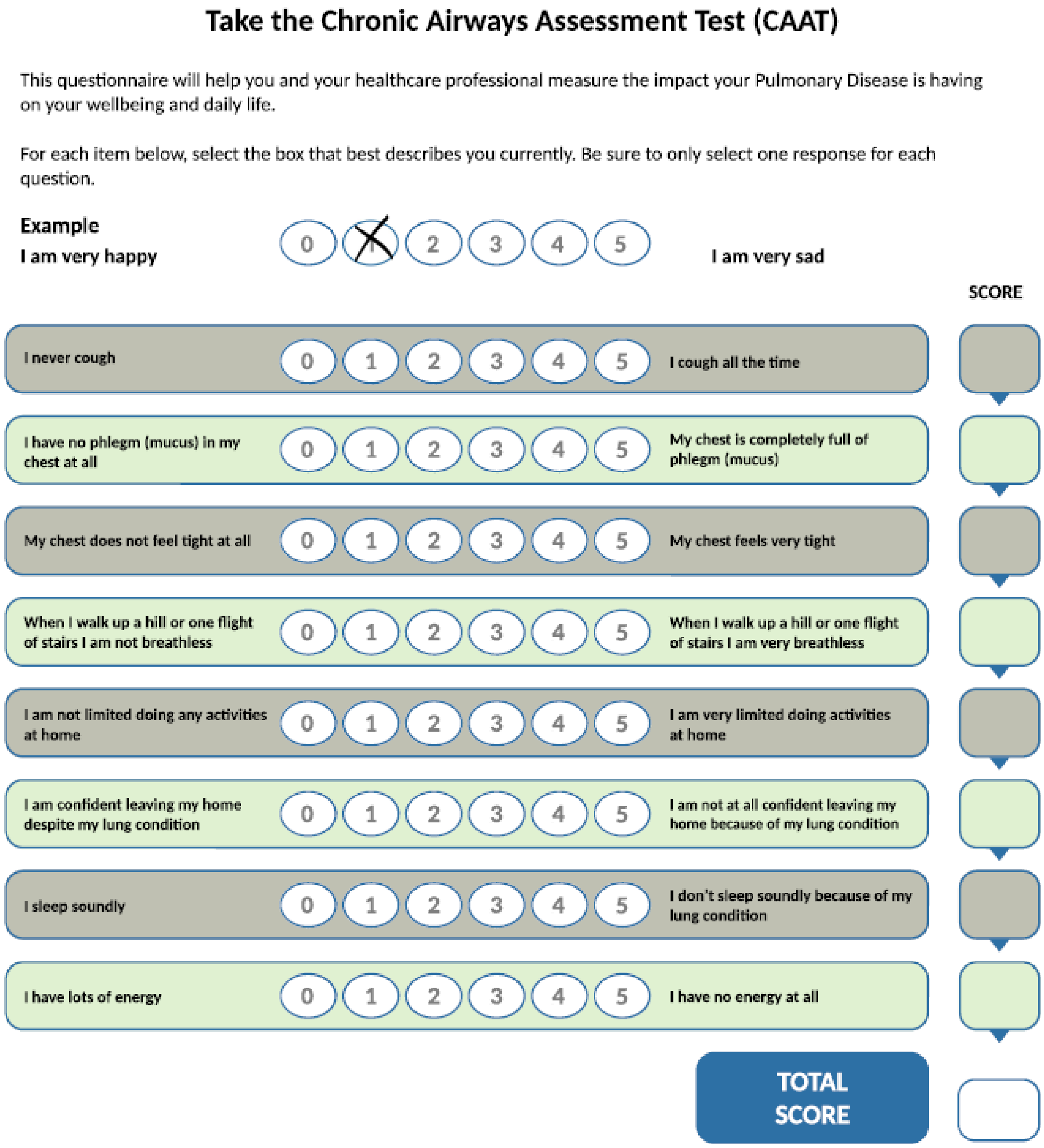

Take the Chronic Airways Assessment Test (CAAT)

This questionnaire will help you and your healthcare professional measure the impact your Pulmonary Disease is having on your wellbeing and daily life.

For each item below, select the box that best describes you currently. Be sure to only select one response for each question.

Example

I am very happy	0	~~1~~ (X)	2	3	4	5	I am very sad	SCORE
I never cough	0	1	2	3	4	5	I cough all the time	
I have no phlegm (mucus) in my chest at all	0	1	2	3	4	5	My chest is completely full of phlegm (mucus)	
My chest does not feel tight at all	0	1	2	3	4	5	My chest feels very tight	
When I walk up a hill or one flight of stairs I am not breathless	0	1	2	3	4	5	When I walk up a hill or one flight of stairs I am very breathless	
I am not limited doing any activities at home	0	1	2	3	4	5	I am very limited doing activities at home	
I am confident leaving my home despite my lung condition	0	1	2	3	4	5	I am not at all confident leaving my home because of my lung condition	
I sleep soundly	0	1	2	3	4	5	I don't sleep soundly because of my lung condition	
I have lots of energy	0	1	2	3	4	5	I have no energy at all	
							TOTAL SCORE	

Reproduced with permission from the CAAT Governance Board [3].

The CAT and the CAAT were developed by an interdisciplinary group of international experts with support from GSK. CAT and CAAT activities are monitored by a supervisory council that includes independent experts, one of which is the chair of the council. CAT, COPD Assessment Test, CAAT, Chronic Airways Assessment Test and the CAT logo are trademarks of the GSK group of companies. ©2022 GSK 'Group of Companies' or its licensor. All rights reserved.

CAAT: Chronic Airways Assessment Test; CAT: COPD Assessment Test; COPD: chronic obstructive pulmonary disease.

SUPPLEMENTARY FIGURE 2 Summary of patients included in the analysis according to physician-assigned diagnostic label

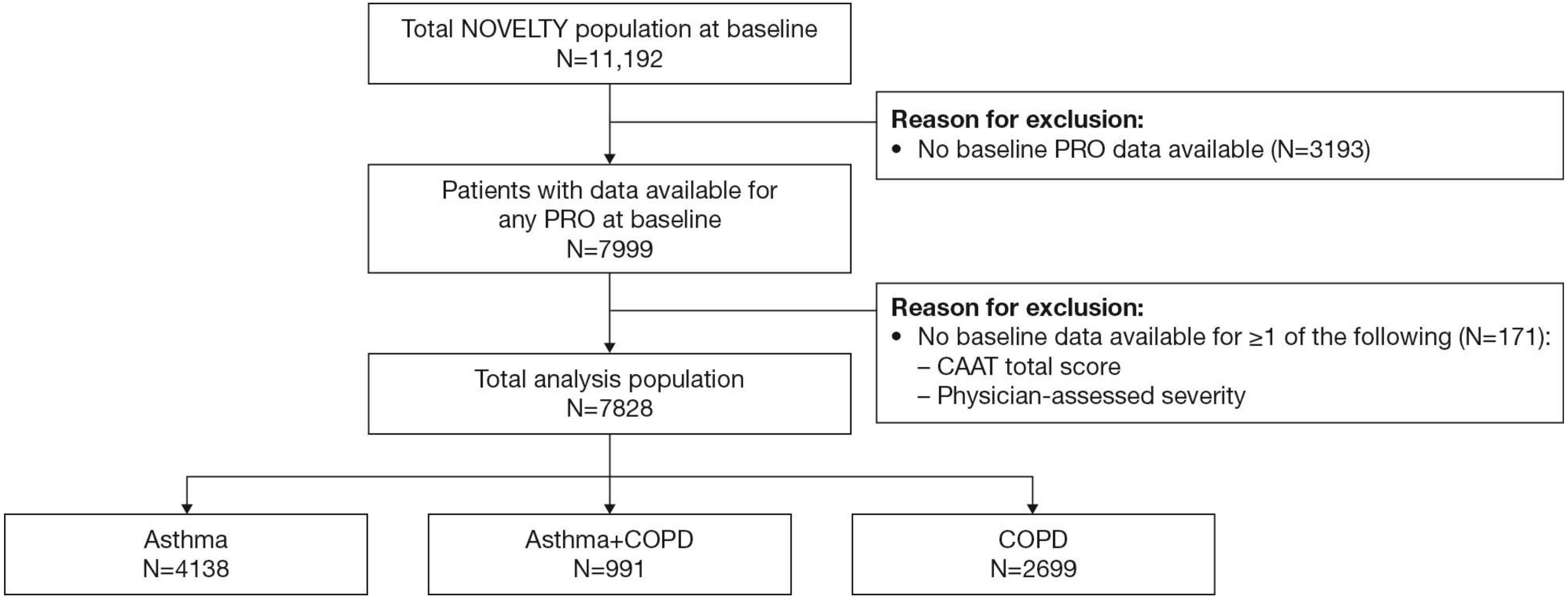

CAAT: Chronic Airways Assessment Test; COPD: chronic obstructive pulmonary disease; N: total number of patients in the group; PRO: patient-reported outcome.

SUPPLEMENTARY FIGURE 3 Histograms showing the distribution of CAAT total scores in all patients by diagnosis and physician-assessed severity

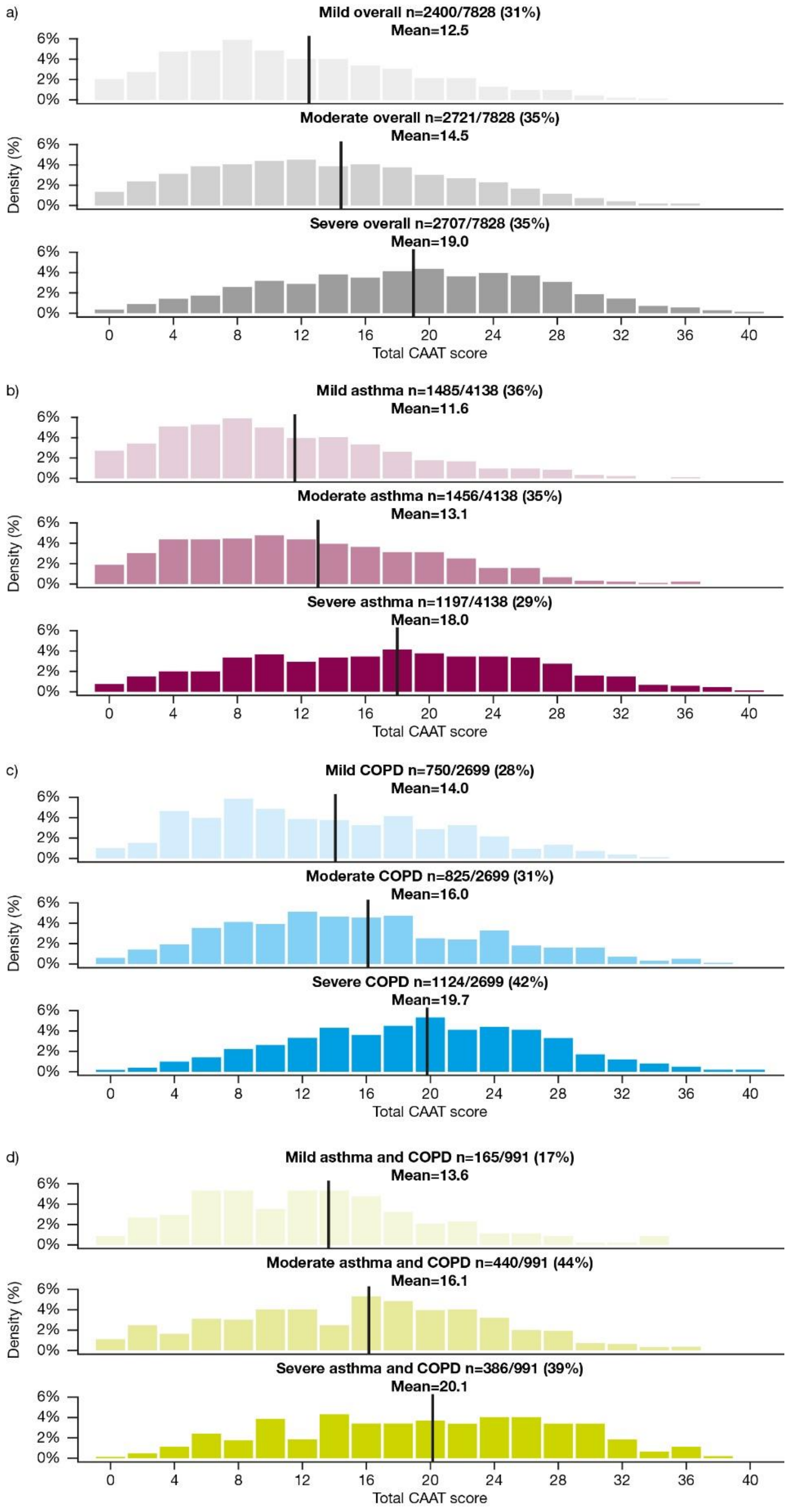

Histograms showing distribution of CAAT total scores. The black line indicates the mean. The density represents the proportion of patients who had a particular CAAT score in the specified diagnostic and severity group. CAAT: Chronic Airways Assessment Test; COPD: chronic obstructive pulmonary disease.

SUPPLEMENTARY FIGURE 4 Association of CAAT score with categorical variables a) physician-assessed severity and b) mMRC dyspnoea grade in patients with asthma, asthma+COPD or COPD: sensitivity analysis adjusted for age

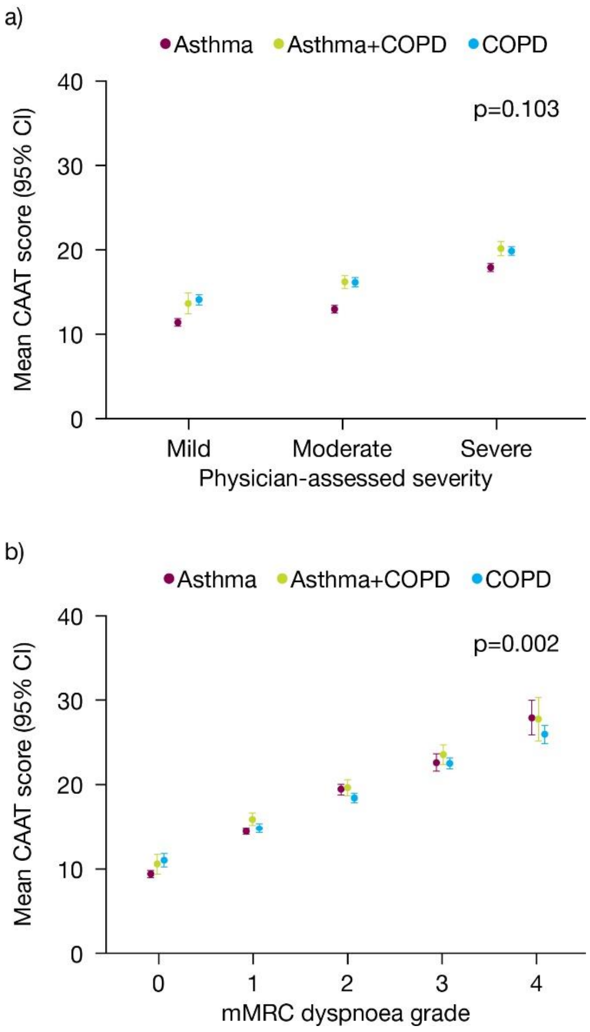

This figure shows interaction plots designed to visualise any differences in the association between CAAT score and each clinical characteristic between diagnostic groups. To test whether the association between CAAT score and each clinical characteristic differed between diagnostic groups, an interaction term was included in each model. Physician-assessed severity and mMRC dyspnoea grade were analysed as categorical variables. CAAT: Chronic Airways Assessment Test; COPD: chronic obstructive pulmonary disease; mMRC: modified Medical Research Council.

SUPPLEMENTARY FIGURE 5 Association of CAAT score with continuous variables a) post-bronchodilator FEV_1 (% predicted) and b) RSQ total score in patients with asthma, asthma+COPD or COPD and c) ACT score in patients with asthma or asthma+COPD: sensitivity analysis adjusted for age

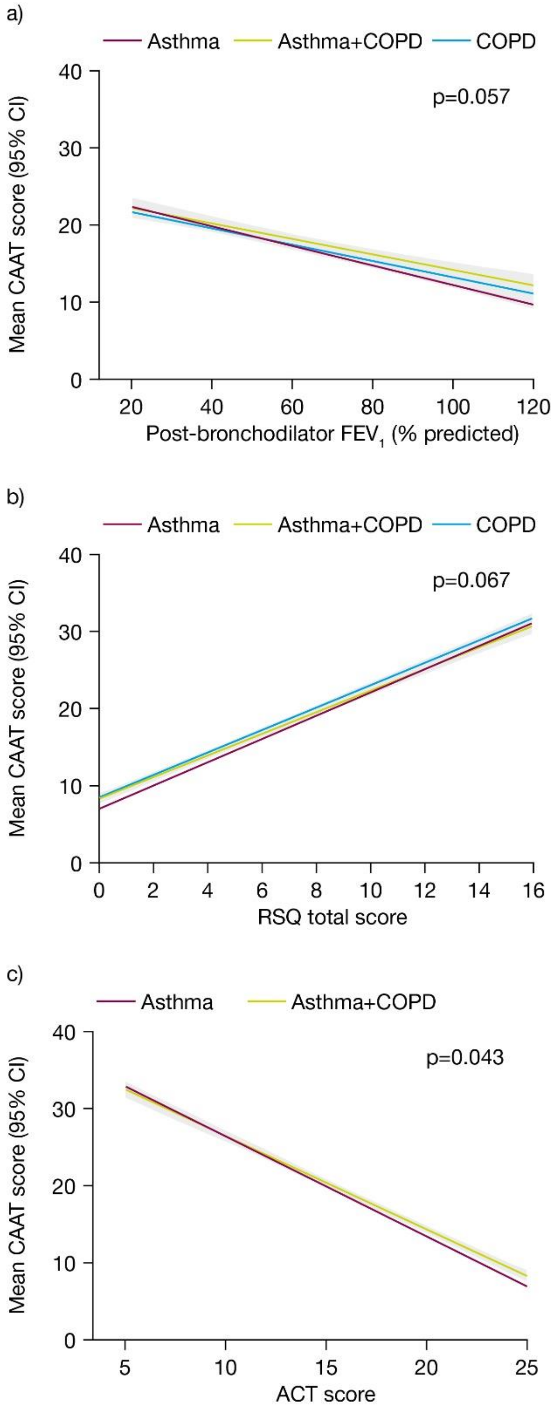

This figure shows interaction plots designed to visualise any differences in the association between CAAT score and each clinical characteristic between diagnostic groups. To test whether the association between CAAT score and each clinical characteristic differed between diagnostic groups, an interaction term was included in each model. Post-bronchodilator FEV_1 % predicted, RSQ total score and ACT score were analysed as continuous variables. Grey bands indicate 95% confidence intervals. ACT: Asthma Control Test; CAAT: Chronic Airways Assessment Test; COPD: chronic obstructive pulmonary disease; FEV_1: forced expiratory volume in 1 second; RSQ: Respiratory Symptoms Questionnaire.

SUPPLEMENTARY FIGURE 6 Association of CAAT score with categorical variables a) physician-assessed severity and b) mMRC dyspnoea grade in patients with asthma, asthma+COPD or COPD: sensitivity analysis adjusted for age, sex and smoking status

a)

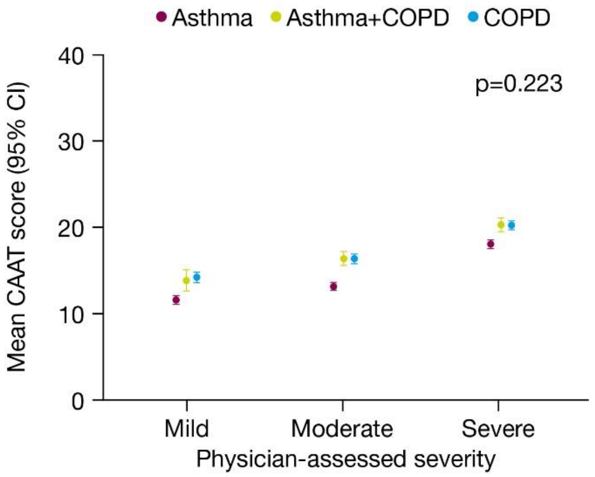

b)

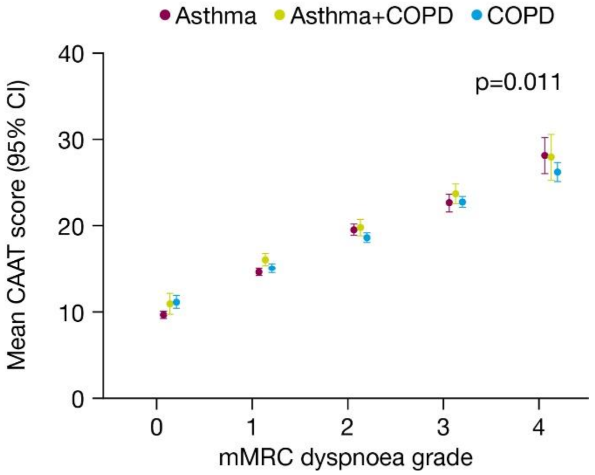

This figure shows interaction plots designed to visualise any differences in the association between CAAT score and each clinical characteristic between diagnostic groups. To test whether the association between CAAT score and each clinical characteristic differed between diagnostic groups, an interaction term was included in each model. Physician-assessed severity and mMRC dyspnoea grade were analysed as categorical variables. CAAT: Chronic Airways Assessment Test; COPD: chronic obstructive pulmonary disease; mMRC: modified Medical Research Council.

SUPPLEMENTARY FIGURE 7 Association of CAAT score with continuous variables a) post-bronchodilator FEV_1 (% predicted) and b) RSQ total score in patients with asthma, asthma+COPD or COPD and c) ACT score in patients with asthma or asthma+COPD: sensitivity analysis adjusted for age, sex and smoking status

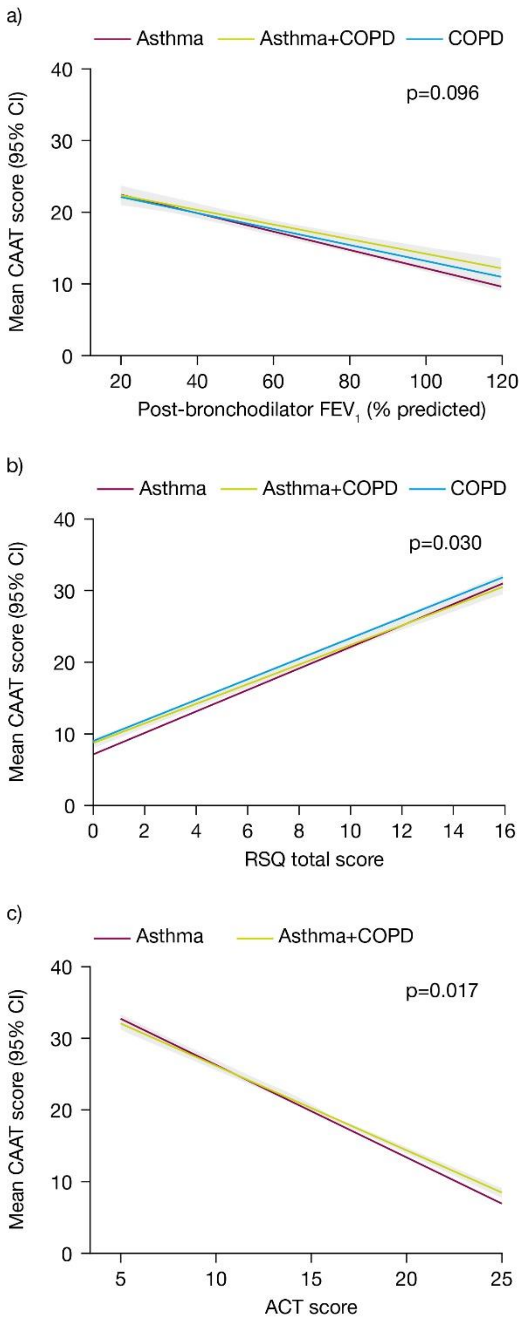

This figure shows interaction plots designed to visualise any differences in the association between CAAT score and each clinical characteristic between diagnostic groups. To test whether the association between CAAT score and each clinical characteristic differed between diagnostic groups, an interaction term was included in each model. Post-bronchodilator FEV_1 % predicted, RSQ total score and ACT score were analysed as continuous variables. Grey bands indicate 95% confidence intervals. ACT: Asthma Control Test; CAAT: Chronic Airways Assessment Test; COPD: chronic obstructive pulmonary disease; FEV_1: forced expiratory volume in 1 second; RSQ: Respiratory Symptoms Questionnaire.

REFERENCES

1. Jones PW, Tabberer M, Chen WH. Creating scenarios of the impact of COPD and their relationship to COPD Assessment Test (CAT) scores. *BMC Pulm Med* 2011;11:42.
2. Schatz M, Sorkness CA, Li JT, *et al.* Asthma Control Test: reliability, validity, and responsiveness in patients not previously followed by asthma specialists. *J Allergy Clin Immunol* 2006;117:549–556.
3. Global Allergy and Airways Patient Platform. The Chronic Airways Assessment Test (CAAT) formerly known as the COPD Assessment Test (CAT). https://gaapp.org/caat-cat/. Date last updated: 2024. Date last accessed: September 17 2024.
